# Action recommendations to support people living in precarious conditions during a pandemic

**DOI:** 10.1093/eurpub/ckac129.464

**Published:** 2022-10-25

**Authors:** Z Öcek, AM Volkmann, V Klünder, A Basil, M Geise, M Coenen

**Affiliations:** 1 Chair of Public Health and Health Services Research, Ludwig-Maximilians-University, Munich, Germany; 2 Pettenkofer School of Public Health, Munich, Germany; 3 Rachel Carson Center, Ludwig-Maximilians-Universität München, Munich, Germany; 4 University College London, London, UK; 5 NIVEL, Utrecht, Netherlands

## Abstract

**Background:**

The COVID-19 pandemic has deepened pre-existing health inequalities. As members of an EU-funded research consortium (SoNAR-Global) tasked with assessing vulnerabilities in five European countries, we explored the impacts of the pandemic on vulnerable populations and identified concrete actions that strengthen resilience. Here, we present our action recommendation development process for populations living in precarious conditions such as refugees, migrants and those living below the poverty line in Munich, Germany.

**Methods:**

The process began by interviewing 82 people who were likely to face vulnerability mechanisms, followed by interviews with 19 community representatives. Based on this qualitative research data, we defined challenges and resilience factors, and matched these with a list of action recommendations. This list, named “Provision of social support to people living in precarious conditions in times of a pandemic”, was considered by a panel with ten experts on migration, health, poverty, social support, and inclusion.

**Results:**

Through discussion, we eliminated one recommendation and re-defined three others and achieved consensus on six recommendations. They are: 1) work towards eliminating opportunity inequity in education, 2) streamline bureaucratic processes to improve access to care, 3) establish structures that allow for continuing social support even during a pandemic, 4) refrain from isolating socially vulnerable groups through blanket pandemic mitigation measures, 5) Hybrid (online and live) solutions for inclusion, 6) improving healthcare empowerment.

**Conclusions:**

Through an inclusive process that involved citizens, vulnerable populations, and health- and government experts we identified a set of actions that should be taken now to ensure an equitable and just approach to pandemic preparedness. Our panel identified feasible and achievable goals within those recommended actions.

**Key messages:**

• Vulnerability mechanisms are multi-layered. Therefore, policies that need to be developed should be based on comprehensive approaches, not selective interventions.

• Actions to mitigate vulnerabilities in pandemics must be ambitious in order to prevent further exacerbation of existing issues with potentially catastrophic long-term consequences.

